# Advancing service offerings in food and nutrition metrology: the updated METROFOOD-RI service chart

**DOI:** 10.3389/fnut.2025.1711663

**Published:** 2025-12-18

**Authors:** C. De Bruyn, S. Sharma, C. Zoani, Joris Van Loco

**Affiliations:** 1 Sciensano, Chemical and Physical Health Risks Department, Brussels, Belgium; 2 Italian National Agency for New Technologies, Energy and Sustainable Economic Development (ENEA), Rome, Italy

**Keywords:** METROFOOD-RI, European research infrastructures, European strategy forum on research infrastructures (ESFRI), food metrology, agrifood, Health & Food, sustainability, service catalog

## Abstract

METROFOOD-RI is a pan-European research infrastructure (RI) dedicated to promoting metrology in food and nutrition, integrating an array of interdisciplinary fields such as agrifood, food safety and quality, traceability, human health, and environmental sustainability. A core element of METROFOOD-RI's implementation is its service chart, including a structured catalog which provides an overview of the services offered across its network of facilities. During the Early Phase Implementation project (METROFOOD-EPI), the consortium undertook a comprehensive update of the service chart, addressing limitations from the Preparatory Phase (METROFOOD-PP) and aligning the service portfolio with current capabilities and user needs. This article presents the context and outcomes of the service chart update in a scientific framework. We describe the rationale for revising the original service chart, and the methodological steps followed to refine service categorization. The updated service chart is organized into four primary categories: Research services, ICT and Data services, Advisory services, and Education and Training services. Within these, the *Research* category is further structured into sub-domains (Agrifood, Metrology tools, Health, Environment & Sustainability) to encompass the RI's broad scope. We detail the definitions and rationale for each category and subcategory, highlighting how this structure integrates services across domains and aligns with key priorities in agrifood innovation, public health, sustainability, and metrology. The updated chart improves internal coordination among METROFOOD-RI partners by aligning resources to identified needs and revealing gaps, and also enhances external user experience by allowing easy navigation of available services through its associated catalog. Finally, we discuss the value of the service chart as a foundation for future tools, such as a METROFOOD-RI online access portal and a membership app, and its role in supporting a multidisciplinary, open science approach.

## 1 Introduction

Modern challenges in food and nutrition research demand high-quality measurements, standardized methods, and interdisciplinary collaboration. METROFOOD-RI, the infrastructure for promoting metrology in food and nutrition, is being established as a distributed European Research Infrastructure Consortium (ERIC) to address these needs (1). It provides high-quality metrology services, knowledge and tools in food and nutrition, across an interconnected range of fields spanning the entire food value chain, from agrifood production and quality assurance to human health and environmental sustainability (2–6). In practice, METROFOOD-RI consists of an integrated network of physical facilities (laboratories, pilot plants, experimental fields/kitchens labs) and an electronic infrastructure (e-RI) that together support scientific excellence in areas such as food quality and safety, traceability and authenticity, environmental monitoring, circular bioeconomy, and nutrition research (7). By integrating the principles of metrology (the science of measurement) into food analysis and related domains, METROFOOD-RI aims to harmonize standards, foster cross-border collaboration, and offer a wide set of services that can be accessed by academia & research, food business operators, policy-makers and consumers for advancing research and innovation in the food and nutrition sector.

A critical step in operationalising such a research infrastructure is to clearly define and organize the services it offers (8, 9). Within the broader ESFRI “Health & Food” domain, several complementary RIs already offer critical services: BBMRI-ERIC for biobanking and biospecimen access (10, 11), ELIXIR for life-science data resources (12, 13), EATRIS for translational medicine support (14, 15), etc. These examples illustrate the range of service typologies, spanning physical access, data management, and training. In this landscape, METROFOOD-RI distinguishes itself by integrating both physical and electronic components to support metrology across the entire agrifood system, from production to consumption, with a strong focus on measurement quality, traceability, and sustainability.

As METROFOOD-RI progressed toward its implementation phase, and in response to evolving scientific priorities, digitalisation trends and policy frameworks such as the European Green Deal and the Farm to Fork Strategy, it became evident that its initial chart required refinement. The Early Phase Implementation (EPI) project of METROFOOD-RI (16) included a dedicated task to update and improve the service chart in line with the RI's evolving scope and stakeholder feedback, as well as the changing external context. The motivation for this update was multifaceted. First, it became important to enhance the clarity and granularity of the service chart to better reflect the full range of services provided. Some descriptions and categories were refined to improve user-friendliness and facilitate easier identification of relevant offerings by external users. Second, the update provided an opportunity to more explicitly integrate areas such as training and education programmes, which are central to METROFOOD-RI's mission of supporting capacity building and knowledge transfer in food and nutrition metrology. Third, the RI's capabilities and partner contributions had expanded since the preparatory phase, with new services (e.g., in data management or emerging food technologies) and updated partner facilities requiring inclusion. Lastly, the revision aimed to improve the accessibility and usability of the associated catalog, ensuring that potential users can quickly navigate and understand METROFOOD-RI's overall service offer. This user-centric goal aligns with open science principles, ensuring that the infrastructure's resources are transparent and easily discoverable (17).

In summary, the context for the service chart update was set by METROFOOD-RI's evolution into an implementation phase and the recognition that a more structured, comprehensive, and accessible service catalog would enhance both internal coordination and external usage of the RI. Internally, an improved chart would help align resources with user needs, identify gaps or overlaps in service provision, and facilitate coordination among the 12 participating countries and numerous institutions. Externally, it would serve as the basis for user-facing tools such as a service portal, enabling scientists, companies, and other stakeholders to easily find services ranging from specialized analytical measurements to expert advisory support. The updated chart also underpins the development of the METROFOOD-RI's e-platform, including its membership app and the external access portal under development, which will organize detailed service information for internal management and open-access user consultation. By updating the service chart, METROFOOD-RI aims to deliver high-quality, relevant, and efficient services that are clearly communicated to its users.

This article details the process, novelties, outcomes, and research contributions of the METROFOOD-EPI service chart update. We first describe the methodological approach taken to revise the service chart, including partner involvement and the rationale behind the new categorization. We then present the results: the new service categorization structure, describing each main category and subcategory, and explaining how this structure better captures the RI's offerings. In the discussion, we examine how the new chart integrates services across traditional domain boundaries and aligns with broader priorities in agrifood systems, metrology, public health, and sustainability. We further position METROFOOD-RI relative to peer ESFRI infrastructures, and also outline the expected benefits for both internal stakeholders (partners, management) and external users, highlighting how the updated chart strengthens METROFOOD-RI's capacity to address emerging scientific and societal challenges.

## 2 Methods

### 2.1 Approach to service chart revision

The METROFOOD-EPI project employed a comprehensive, participatory approach to update and refine the METROFOOD-RI service chart. The process built upon the prior framework from the preparatory phase and actively engaged the consortium's partners to ensure the updated chart reflects current capabilities and strategic goals. Service categorization was also informed by reviewing the service charts and catalogs of other ESFRI RIs in the Health & Food domain (e.g., BBMRI-ERIC, ELIXIR, EATRIS), drawing inspiration from best-practice elements such as data interoperability tags and dedicated training modules. At the outset, the team evaluated the initial service chart (18) and related feedback. This included analyzing survey results collected during METROFOOD-PP, where users and partners had previously provided input on available services and their categorization. The evaluation highlighted specific areas in need of refinement (e.g., ambiguities in category definitions, services missing or underrepresented). These insights provided a valuable foundation for restructuring the chart in a way that better reflects the infrastructure's multi- and inter-disciplinarity nature and user expectations.

### 2.2 Partner involvement and data gathering

Recognizing that METROFOOD-RI's strength lies in the diverse expertise of its national nodes, the update process was highly inclusive. Each partner institution was invited to reassess and update its service offerings. A structured template and survey (developed using tools such as MIRO board and LimeSurvey) were distributed to capture detailed input from all partners. Partners were asked to list their services, describe them, and, importantly, propose how they should be categorized. They were also encouraged to suggest their own categorization terms based on their specific expertise, ensuring that the resulting structure would be meaningful across disciplines.

The collected partner inputs were then consolidated and analyzed by the task coordination team (led by Sciensano, Belgium). First, synonymous or closely related category labels were clustered and normalized to a single preferred term to remove terminological variance across partners. Next, frequency counts were computed across partners for each label. Main categories were retained when they showed broad consensus across partners, while subcategories were selected when they appeared frequently and were then placed under the relevant main category. Proposals with limited uptake were either nested under the most relevant main category or flagged for future consideration if no clear alignment was evident. Through this process, certain common themes and frequently suggested categories emerged. For example, many partners independently identified services related to food quality and authenticity, or to data analysis, or training activities, indicating the need for those as distinct grouping concepts. Then, an iterative and collaborative approach was adopted, with successive rounds of consultation and optimisation within dedicated workshops, leading to a shared categorization framework. This ensured that each partner could see their services fitting naturally into the new chart, and the categories reflected the diversity of the METROFOOD-RI community. The result was a consensus-based structure that balances scientific coherence with practical usability.

### 2.3 Refinement and validation

With a draft categorization, the team integrated the updated information on individual services (provided by partners) into the new structure. Services were grouped according to the agreed categories, and any ambiguities were resolved through direct communication with the providing partners or via smaller working group meetings. If a service could potentially fall under multiple categories, the decision was made based on the service's primary purpose and the perspective of likely users. In some cases, cross-tagging of subcategories was applied to reflect the interdisciplinary nature of certain services.

The refined chart was then validated by cross-checking with METROFOOD-RI's objectives and current activities. Particular attention was given to ensuring alignment with strategic goals and broader scientific and policy frameworks, including European and international research agendas (ESFRI, EFSA, EOSC, FNS-Cloud, etc.). For example, categories were evaluated for how well they map to priorities such as food chain transparency, emerging food risks, sustainability and circular bioeconomy, and innovation in food technology. This approach also enabled the identification of a set of integrated services, cross-cutting service offerings combining multiple capabilities to address complex R&I themes. These integrated services helped shape the categorization by highlighting meaningful combinations of expertise that should be easily navigable within the chart.

After internal review and minor adjustments, the updated service chart was finalized and documented in Deliverable D4.3 (Service Chart Update) (16) within the METOFOOD-EPI project. The final structure and definitions were disseminated to all partners for implementation, and each national node now uses the new categories when describing its services in the METROFOOD-RI information systems.

## 3 Results: the updated service chart structure

### 3.1 Overview of the new categorization

The revised METROFOOD-RI service chart is organized into four core categories, designed to cover all key areas of the infrastructure's capabilities:

- Research services
- ICT & Data services
- Advisory services
- Education & Training services

**Figure 1:**
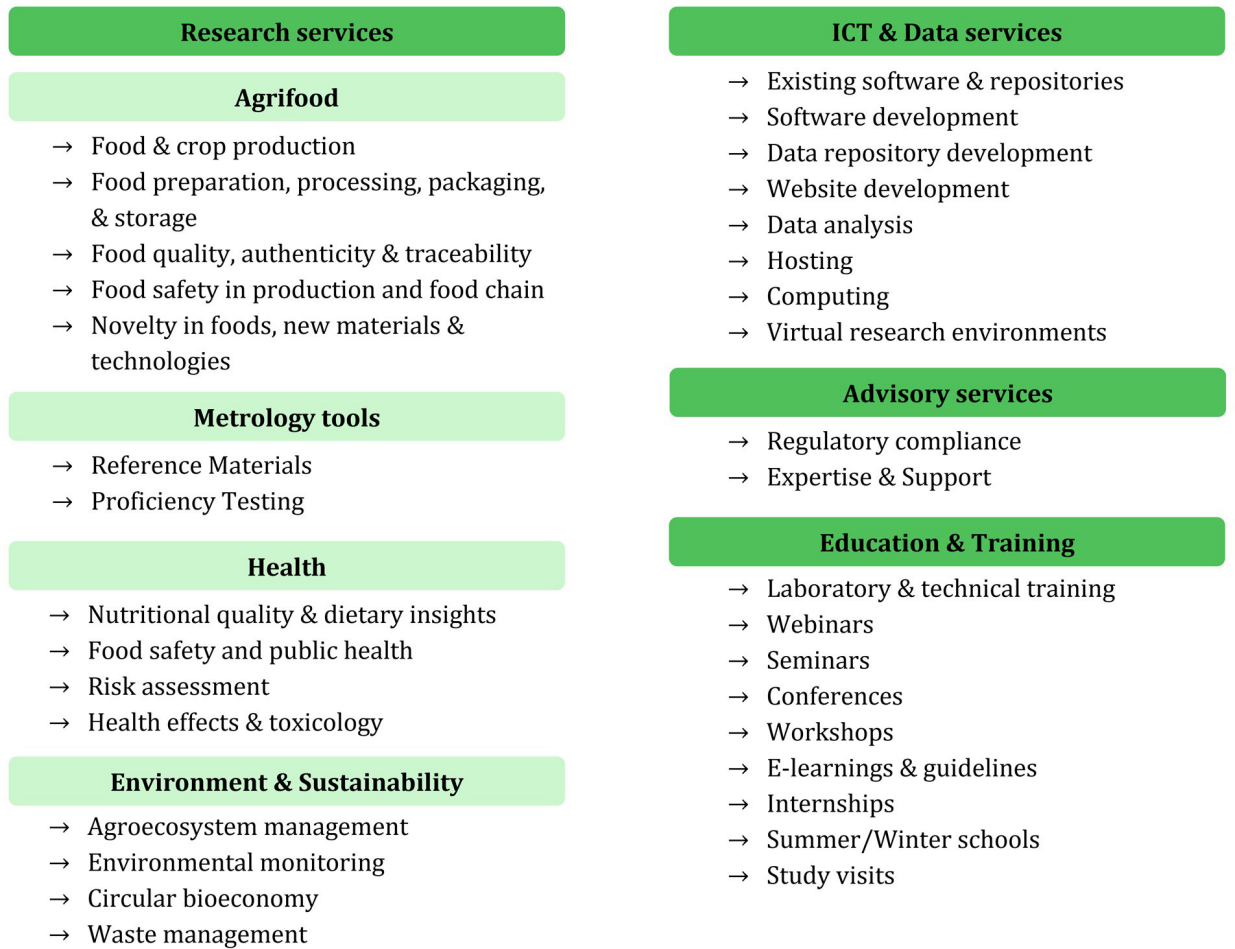
The METROFOOD-RI updated service categorization, illustrating the four top-level service categories and their subcategories. Research services are subdivided into four thematic domains: Agrifood, Metrology tools, Health, and Environment & Sustainability, each of which contains specific subcategories (bulleted). The ICT & Data, Advisory, and Education & Training categories have their respective subcategories listed without intermediate groupings.

Each of these primary categories is further divided into subcategories that add specificity, thereby enabling users to navigate the complex range of services with greater ease. Given its central role within METROFOOD-RI, the **Research services** category includes an additional layer of organization structured into four thematic sub-domains: Agrifood, Metrology Tools, Health, and Environment & Sustainability. The service categorization is presented in Figure 1, followed by detailed information of each of the categorization terms in the next section and detailed definitions in **Annex I**.

As shown in Figure 1, the *Research services* category is structured to reflect METROFOOD-RI's extensive scientific activities across the food and nutrition domain. Within *Research services*, four major thematic areas were identified:

- **Agrifood**, covering the entire food supply chain and agrifood system;
- **Metrology tools**, covering measurement and quality assurance tools specific to food analysis;
- **Health**, covering food, nutrition, and health interactions (including safety and public health);
- **Environment & Sustainability**, covering environmental aspects and sustainable practices in food systems.

Each of these intermediate sub-domains contains several more granular subcategories to capture the range of research services offered. This multi-tiered structure was designed to reflect the diversity of METROFOOD-RI's research portfolio and support intuitive navigation for users with different backgrounds and needs. They include experimental studies, advanced analytical testing, methodological developments, and scientific evaluations. These services play a key role in driving innovation, supporting evidence-based policymaking, and fostering technological innovation. By addressing complex challenges and bridging multiple disciplines, these services generate new knowledge and catalyse interdisciplinary collaboration across scientific and industrial domains.

Within the **Agrifood** research sub-domain, METROFOOD-RI groups services that span the entire food production and supply chain. This includes services related to primary production, food processing and preservation, packaging and storage, as well as assessment of food quality, safety, authenticity, and traceability. *Agrifood research* services even extend to the development of novel foods, materials, and technologies aimed at improving the food system. Subcategories under Agrifood (see Figure 1) include *Food & crop production, Food preparation, processing, packaging & storage, Food quality, authenticity & traceability, Food safety in production and the food chain*, and *Novel foods, new materials & technologies*. Each of these represents a cluster of services. For instance, *Food quality, authenticity & traceability* may include services focused on compositional analyses, authenticity testing via isotopic or metabolomic methods, and supply chain transparency tools. This structure ensures that research services addressing agrifood innovation and integrity are easily identifiable.

The **Health** sub-domain of *Research services* encompasses those activities at the center of food/nutrition and human health. This includes services focused on *nutritional quality and dietary insights* (e.g. nutrient profiling, bioavailability studies), *food safety and public health* (such as pathogen detection, contaminant risk assessment), as well as *toxicology and health effect studies* related to food compounds. For example, a METROFOOD-RI partner offering metabolomic analysis for food safety or clinical studies on nutrition and health, would classify these under the *Health* category. By explicitly including *Health* as a category, the service chart aligns with the One-Health perspective (28) and acknowledges the importance of nutrition and food safety in protecting consumer health. It also reflects policy priorities on healthy diets and the prevention of food-related diseases. Any service that primarily deals with the impact of food on human health or the assessment of health risks in the food chain is labeled under this sub-domain.

The **Environment & Sustainability** sub-domain covers services that relate to environmental monitoring, ecosystem management in food production, and the sustainability of agrifood systems. Subcategories include *Agroecosystem management* (e.g., sustainable farming practices, soil health, agro-biodiversity), *Environmental monitoring* (e.g. monitoring of pollutants or greenhouse gas emissions), *Circular bioeconomy* (e.g., valorisation of food by-products, waste reduction technologies), and *Waste management*. This category reflects METROFOOD-RI's commitment to supporting the European Green Deal (19) and Sustainable Development Goals (20) through its services. Many partners provide services in this domain, such as analyses of environmental contaminants, lifecycle assessments of food processes, or technologies for converting food waste into value-added products. By grouping these under a dedicated category the chart ensures visibility of services supporting environmentally sustainable food systems.

A notable design decision was to incorporate **Metrology tools** as a sub-domain within *Research services*, rather than as a separate top-level category. This reflects the principle that metrology is not an isolated activity but a foundational element across all research domains. *Metrology tools* include services such as the development and provision of Reference Materials (RMs), Proficiency Testing (PT) schemes, calibration services, and advanced analytical method development. Essentially, through these services the infrastructure ensures accuracy, precision, and standardization in measurements, which are foundational for reliable and reproducible research outcomes. Categorizing them under *Research services* emphasizes their cross-cutting role. These offerings support harmonization and mutual trust in data across laboratories and institutions, a core objective of METROFOOD-RI.

Moving to the other primary categories, **ICT & Data services** form the seconds pillar of the service chart. These services are crucial for building the e-infrastructure component of METROFOOD-RI. They encompass the digital tools, platforms, and expertise required to handle data and computations in food and nutrition research. *ICT & Data services* support data-driven decision-making by providing the tools and platforms to collect, manage, analyse, and share data effectively. This category includes subcategories such as *Existing software & repositories* (e.g., databases of food composition or metrology data that METROFOOD-RI makes available), *Software development* (custom software or apps for laboratory management, data processing, etc.), *Data repository development* (services to build and host new databases or data portals), *Website development* (for scientific web platforms), *Data analysis* (expert services in statistical analysis, machine learning on food data), *Hosting* (providing computational infrastructure or cloud services for food data), *Computing* (high-performance computing or simulations for food research), and *Virtual research environments* (integrated online platforms where researchers can collaborate and use data/tools remotely). By explicitly listing these services, the chart recognizes that information technology and data management are now inseparable from experimental research. The *ICT & Data category* signals to users that METROFOOD-RI not only provides physical lab services, but also digital services such as data platforms and analysis support. This is particularly valuable for enabling open data and data sharing among stakeholders, thereby promoting transparency and collaboration.

The **Advisory services** category encompasses the consultative and support services that METROFOOD-RI offers. These services leverage the expertise of the RI's scientists and institutions to guide and assist stakeholders. Advisory services typically focus on providing essential guidance and technical expertise to address specific challenges, for example, helping a food company navigate regulatory requirements or offering expert advice on method validation. In the updated chart, two subcategories capture the main types of advisory services: *Regulatory compliance* and *Expertise & Support*. *Regulatory compliance* services include advising on food regulations, standards, and quality systems, such as ISO standards for laboratories, food safety regulations like HACCP, labeling laws, etc. *Expertise & Support* is a broader subcategory that covers technical consulting, problem-solving, and customized support. These advisory offerings are particularly useful for industry stakeholders (like SMEs in the food sector) or policy makers who might need expert input on food measurement issues. In the service entries, advisory services are often indicated alongside research services, e.g., a given service entry might list categories “Research (Agrifood); Advisory (Regulatory compliance, Expertise & support)” to show it provides both experimental analysis and advisory guidance. This dual listing is now clearly supported by the chart's structure.

Finally, **Education & Training services** constitute the fourth category, reflecting METROFOOD-RI's commitment to developing human capital and disseminating knowledge. This category includes a wide range of formal and informal learning opportunities aimed at building competencies and skills in food and nutrition metrology. Subcategories include: *Laboratory & technical training, Webinars, Seminars, Conferences, Workshops, E-learnings & guidelines, Internships, Summer/Winter schools*, and *Study visits* (Figure 1). These offering cover everything from hands-on training in laboratory techniques to virtual educational sessions and exchange programmes. *Education & Training services* ensure continuous learning and skill development, offering diverse and flexible learning opportunities to stakeholders so that they can stay abreast of the latest methods and best practices. A partner might also host *webinars* on specialized topics (e.g., uncertainty in nutritional analysis), organize *workshops* or *conferences* that bring together experts and users (contributing to community building and dissemination), or provide *e-learning modules and guidelines* accessible online. By having a dedicated category, the service chart explicitly showcases these capacity-building services. This will help external users, such as students, professionals, or researchers seeking training, to identify learning opportunities offered by METROFOOD-RI, while also encouraging internal coordination in developing new training modules and avoiding duplication. *Education & Training services* complement the technical services even, ensuring that knowledge is shared and that users have the know-how to effectively engage with the infrastructure.

### 3.2 Integrated services across domains

**Figure 2:**
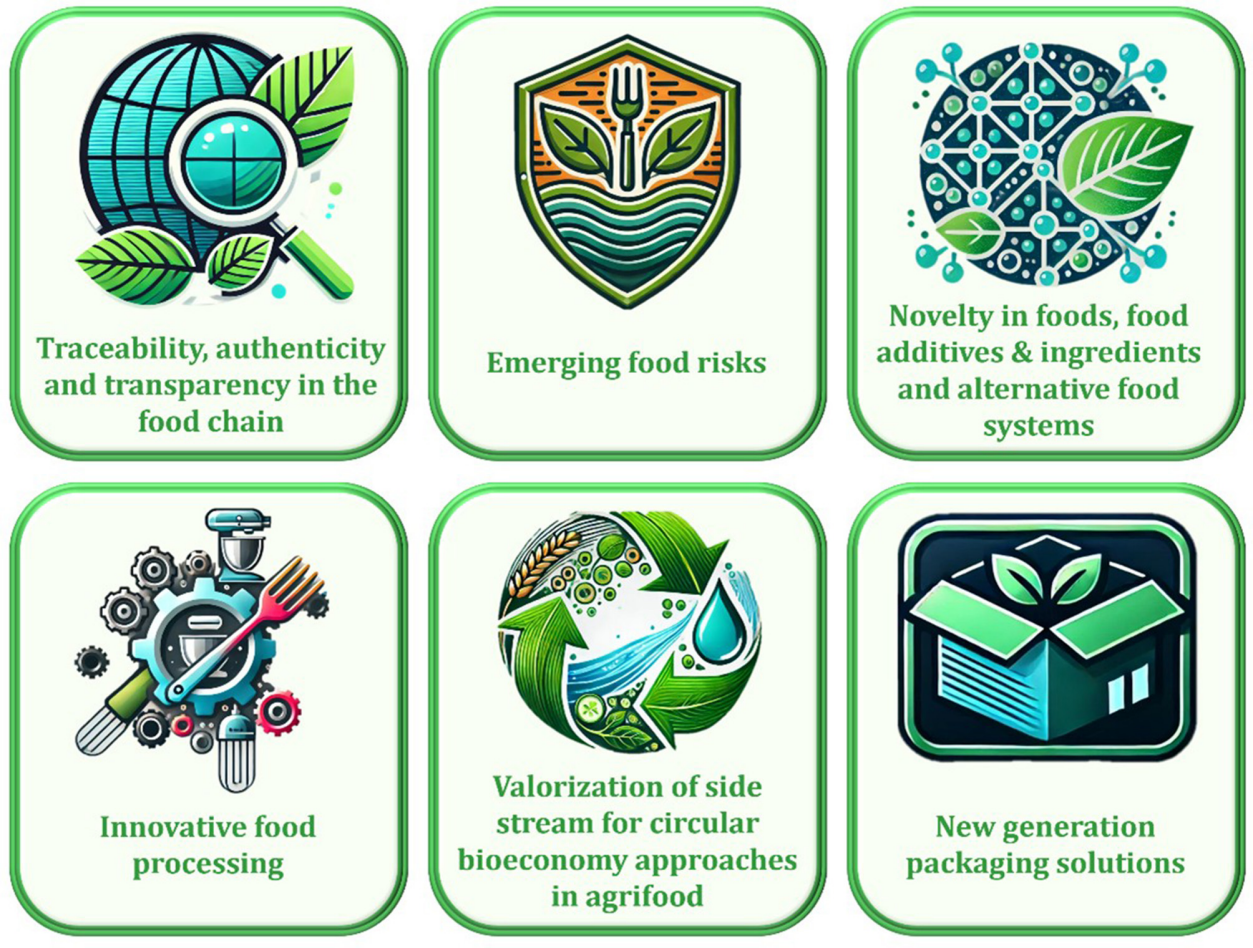
METROFOOD-RI integrated services.

The service chart update actively fosters an integrated approach to food and nutrition research services. One manifestation is the identification of **Integrated services** that combine multiple capabilities across categories, realizing a pipeline of multiple services to address complex and cross-cutting topics. Six integrated service themes were therefore identified as particularly relevant: (1) *Traceability, authenticity, and transparency in the food chain*, (2) *Emerging food risks*, (3) *Novelty in foods, additives & alternative food systems*, (4) *Innovative food processing*, (5) *New generation packaging solutions*, and (6) *Valorisation of side streams for circular bioeconomy*, visualized in Figure 2. Detailed definitions of each integrated service are available in **Annex I**. These themes reflect pressing challenges in the agrifood and require contribution from multiple domains spanning research, data, advisory, and training dimensions. For example, the theme *Emerging food risks* might involve analytical services for contaminant detection (Research: Health/Agrifood), digital tools for risk prediction (ICT & Data), expert guidance on risk mitigation strategies (Advisory), and training food safety professionals (Education & Training). The updated chart makes it easier to assemble such integrated offerings by clearly tagging services across those needed dimensions. This integrated perspective enhances the visibility and usability of METROFOOD-RI's multidisciplinary potential, allowing users to access bundled services that address real-world challenges holistically. It also supports internal coordination by highlighting synergies among partners and encouraging collaborative service development. By enabling thematic clustering, the chart provides a flexible framework for future expansion and innovation.

### 3.3 Internal consistency and improvements

The updated service chart now serves as a single, consistent reference for METROFOOD-RI's service offer. In total, the chart organizes over 200 distinct services across all partner nodes integrated into a searchable catalog. Each service entry in the catalog includes information such as the providing institution, a description of the service, target users, access modes (physical, remote, virtual, or hybrid access), and the relevant service categories. The essential information forming the basis of the service chart and catalog was carefully selected to ensure clarity, usability, and alignment with user needs.

**Figure 3:**
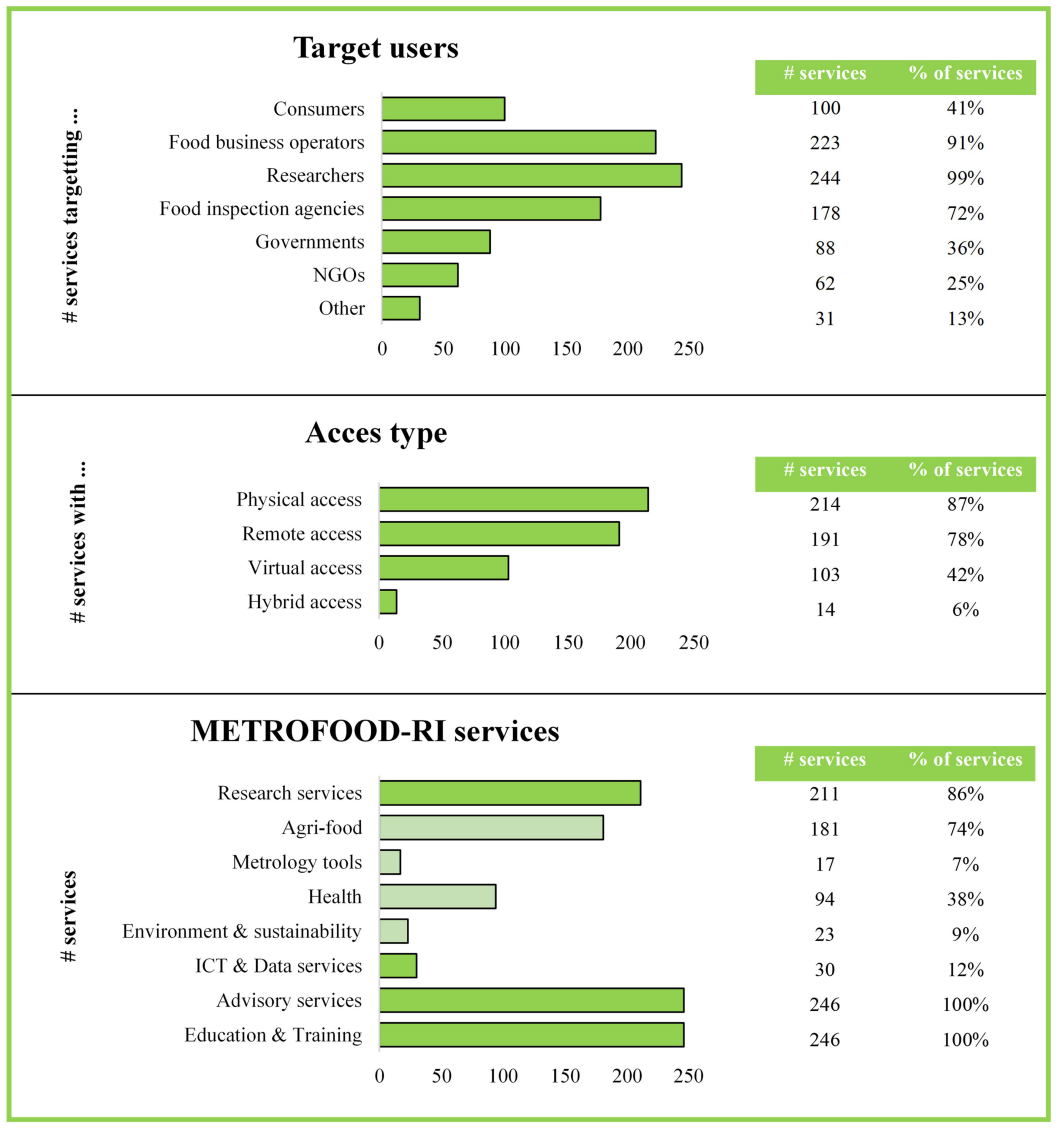
Quantified overview of the target users, access types, and specific service categories of all METROFOOD-RI services (total of 246 services in 2024).

A quantified overview is provided in Figure 3, illustrating the distribution of the target users, access types and service categories of all 246 services currently available within METROFOOD-RI. Results show that the services most frequently target researchers (99%) and food business operators (91%), consistent with the prominence of the Research category (86% of services), which reflects METROFOOD-RI's laboratory and analytical focus. The ICT & Data category accounts for 12%, evidencing e-infrastructure capacity, while all services will provide Advisory and Education & Training services, demonstrating provision for knowledge transfer and capacity building. In terms of access, physical access predominates (87%), with remote/virtual and hybrid modes providing complementary routes to services.

From a user's perspective, the clarity of the updated structure will directly translate to an enhanced user experience. The service chart underpins the user-facing catalog and access portal currently under development. Users entering the portal will be able to browse or search services by these intuitive categories. For example, a user interested in training can go straight to *Education & Training services*; one interested in obtaining reference materials can look under *Metrology tools*; a researcher interested in nutritional analysis can check under *Health-related research services*, and so forth. This categorization allows users to quickly find relevant offerings without navigating through unrelated entries, which is especially valuable given the broad thematic scope of METROFOOD-RI.

Internally, the chart supports more efficient resource alignment and strategic planning. By reviewing services under each subcategory, partners and managers can identify missing capabilities (to be developed) or areas with overlapping services (that may benefit from coordination or specialization). This structured overview enables evidence-based decisions on service development and collaboration.

Moreover, the service chart will feed into the METROFOOD-RI membership app, the internal platform used by partners for supporting internal management. The membership app uses the same categories to organize detailed service information, making it more straightforward for partners to update their offerings and for the RI management to monitor availability and coverage. This way, the chart acts as a common language across the consortium, streamlining communication and ensuring consistency. If a new service is proposed, partners can easily classify it under the existing categories, reducing ambiguity and errors in service listings.

In conclusion, the updated service chart provides a comprehensive, multi-layered categorization that addresses previous limitations. It successfully incorporates education and training, delineates ICT & data services as a separate category, and refines research service groupings to align with METROFOOD's interdisciplinary mandate. The next section will further discuss the implications of this new structure, including how it aligns with external priorities and supports the infrastructure's future evolution.

## 4 Discussion

### 4.1 Alignment with agrifood, metrology, health, and sustainability priorities

The four primary categories and sub-domains were deliberately designed to mirror critical domains in food and nutrition research and innovation. By having explicit labels for **Agrifood**, **Metrology tools**, **Health**, and **Environment & Sustainability** within the service chart, METROFOOD-RI ensures that its services are framed around strategic priorities relevant to both scientific and policy communities. This alignment has multiple benefits.

First, it makes it easier to communicate METROFOOD-RI's relevance to high-level initiatives. For example, agrifood and sustainability are central to Europe's agenda for a sustainable food system, as articulated in strategies like Farm-to-Fork (21). Metrology and quality underpin the assurance of data needed for all scientific and regulatory efforts (22). Health and nutrition tie into public health objectives and initiatives to improve diet and food safety (23, 24). The service chart now effectively maps METROFOOD-RI's capacities to these themes. As noted, the infrastructure comprises an important cross-section of highly interdisciplinary and interconnected fields throughout the food value chain. The updated categorization directly echoes this cross-section. By framing services in these terms, METROFOOD-RI can better position itself in funding calls, collaborations, and policy dialogues, demonstrating how its work contributes to solving pressing challenges.

Second, alignment makes the infrastructure more understandable and attractive to users from different sectors. Consider a stakeholder from a health agency, seeing a *Health* category and subcategories like *food safety* and *toxicology*, gives them a clear entry point to services relevant to their mission. Similarly, a sustainability-focused NGO or researcher will immediately recognize the *Environment & Sustainability* category as relevant to them. This lowers the barrier for engagement. By using broad domain terminology, we speak the language of users who might not be metrology experts themselves but are looking for solutions to domain-specific problems.

### 4.2 Integration and interdisciplinarity

The structure of the service chart was deliberately designed so that services can be multi-tagged with multiple relevant categories/subcategories. For instance, the service *Minimizing food processing contaminants*, provided by one of the partners, involves a pilot to reduce formation of contaminants during food processing. In the new chart, this service is categorized under *Research* with both *Agrifood* and *Health* subdomains because it addresses food processing (agrifood) and food safety (health), and also under *Advisory* (if it includes guidance on best practices), and *Education & Training* (if it involves a training component). Such multi-faceted tagging mirrors the real-world interdisciplinary nature of food systems problems. It ensures that a user browsing by the *Agrifood* category or by the *Health* category would both discover this service. The updated chart's flexible categorization thus supports a systems approach and allows one service to be visible in all relevant contexts.

Another point on integration is the role of the *ICT & Data* category in bridging activities. Data services enable the sharing and synthesis of results from diverse research services, thus integrating knowledge. For instance, a data repository might combine results from agrifood experiments and health studies, making them interoperable. By elevating *ICT & Data* to a top-level category, we signal that digital integration (such as linking databases or creating common analysis platforms) is a priority. In fact, one partner's ICT service explicitly aims to connect catalogs to catalogs and build interoperability across data systems. This will help unify METROFOOD-RI's outputs and allow meta-analyses that span multiple domains.

The service chart update also identified *Integrated service* clusters that bundle multiple services across categories to tackle complex issues. For example, an integrated service concept named *Traceability, authenticity, and transparency in the food chain* was proposed, which would involve research services (analytical measurements for authenticity), digital traceability tools under ICT & Data, as well as advisory components (guidance on transparency standards). Others include themes like *Emerging food risks* (linking health risk assessment research, rapid analytics, and advisory on risk management) and *Valorisation of side streams for circular bioeconomy* (linking environment & sustainability research with innovation advisory on waste streams). These illustrate how the new chart encourages *seeing connections between services* that were previously listed separately. By integrating such domains, METROFOOD-RI can create multidisciplinary service “packages” to address broad challenges, thereby increasing the impact and user appeal of the infrastructure's offerings.

The integration across domains also promotes interdisciplinary research and innovation. METROFOOD-RI's community includes food chemists, microbiologists, metrologists, data scientists, nutritionists, agronomists, etc. The updated chart encourages these experts to see how their work connects. For example, a chemist providing reference materials (metrology) can see that those reference materials support colleagues working on nutrition (health) or environmental contaminants (sustainability). This fosters a sense of a shared mission rather than isolated tasks. It can inspire collaborative projects within METROFOOD-RI where services from multiple categories are combined to deliver new insights. In essence, the chart is as much a communication and coordination tool as it is a classification tool.

### 4.3 Positioning relative to other ESFRI RIs

Against peer research infrastructures within the Health & Food RI landscape (9), METROFOOD-RI is distinguished by a single coherent classification system that treats laboratory, digital, advisory and training services as co-equal, and integrates physical and electronic services. It provides services across different domains (agrifood, metrology, health, environment) and explicitly supports integrated services that chain capabilities across main categories. The chart further reflects METROFOOD-RI's distinctive focus on food and metrology across the value chain. In comparison, BBMRI-ERIC (biobanking) offers a mature catalog centered on sample/data access and quality management (10, 11). METROFOOD-RI shares this quality ethos (e.g., reference materials, proficiency testing) while extending beyond biospecimens to measurement services and elevating Advisory and Education & Training as first-class categories to support uptake across the agrifood system. ELIXIR (life-science data) is an e-RI focused on data resources, computing and training (12, 13). METROFOOD-RI includes a comparable ICT & Data category but couples it directly with laboratory and pilot facilities, and with advisory/training, enabling end-to-end pathways from experimental need to data reuse and capacity building. EATRIS (translational medicine) provides strong preclinical/biomarker/clinical/regulatory services (14, 15). Similarly, METROFOOD-RI exposes advisory capacity, but its chart is oriented to food and nutrition metrology, linking contaminants, authenticity, processing innovation and sustainability to metrological tools and training. Highly specialized platforms such as INSTRUCT-ERIC (structural biology) and INFRAFRONTIER (mouse models) provide deep vertical capabilities (25, 26), while METROFOOD-RI also emphasizes horizontal integration across diverse food-system use cases.

### 4.4 Improved accessibility and user engagement

A primary objective of the update was to enhance the user-friendliness of the service catalog. The revised categories and subcategories are designed to function as an intuitive menu or taxonomy, helping users to locate services more efficiently and gain a clearer overview of METROFOOD-RI's offerings. Each category is accompanied by a descriptive definition (as partially quoted in the results above), which should promote consistency in how partners describe their services and how users understand these classifications.

From a user engagement standpoint, the updated chart is intended to better showcase METROFOOD-RI's full suite of capabilities so that potential users appreciate the infrastructure's multidisciplinary nature. Although this cross-service visibility has not yet been empirically tested, the new structure is expected to facilitate such “cross-pollination” of engagement.

Similarly, by basing the forthcoming online service portal on this taxonomy, it is anticipated that search engine discoverability will improve: public-facing pages organized under clear headings (e.g. “Research services in Food Safety” or “Training workshops in Food Metrology”) should align more closely with user search queries. As open science and open-access practices continue to gain momentum, making a transparent, well-structured catalog available is likely to increase METROFOOD-RI's visibility across diverse user groups, even as formal usability testing is planned for the portal's launch phase.

Finally, the updated chart is expected to support internal users, such as early-career researchers or technicians within partner institutes, in identifying training and collaboration opportunities across the network. In particular, the *Education & Training* category has been structured to encourage internal knowledge exchange and mobility, which in turn should strengthen the RI's collective expertise and cohesion.

### 4.5 Strategic planning and future directions

The service chart provides a solid foundation for strategic planning as METROFOOD-RI moves toward its operational phase. Its structured categorization enables ongoing monitoring of service coverage and evolution. To support continuous improvement, assess the effectiveness of the service chart in promoting access and collaboration, and underpin long-term sustainability, an annual monitoring cycle will be established, tracking metrics. Key Performance Indicators (KPIs) for this purpose include: user access requests and service usage, user engagement across sectors and categories, end-user satisfaction on service discovery, and participation in training initiatives. These metrics will help identify strengths and areas for refinement, guiding adjustment of category definitions, reinforcement of service areas, or enhancement of user interface design. They will further support evidence-based resource allocation that aligns with strategic priorities, ensures value for stakeholders, and prevents unchecked growth of low-demand services (27).

The service chart update also supports other implementation tasks within METROFOOD-RI, such as the development of the access portal and the implementation of the access policies and procedures, as well as the business model. Its clarity and comprehensiveness will support METROFOOD-RI in planning its service access provisions and user documentation, even providing useful content for promotional and communication materials, contributing to a coherent and transparent service offer.

Despite the significant improvements, some challenges remain. Ensuring that new services are correctly categorized will require ongoing curation. Because some services span multiple categories, thoughtful tagging is required to avoid confusion. Clear definitions and examples already help mitigate this, but additional guidance (or even an ontology) could be developed so that tagging is done thoughtfully. Furthermore, METROFOOD-RI will likely establish a procedure (possibly via the membership app) where any new service entry or any change must be tagged with the appropriate categories from the defined list. The governance might include a small committee or the data management team to oversee this consistency. To further improve interoperability, METROFOOD-RI will adopt common metadata, controlled vocabularies, run metadata checks, and expose open APIs.

Looking forward, the service chart offers potential for collaboration and integration with other infrastructures and platforms, and with the European Open Science Cloud (EOSC). This could further facilitate interdisciplinary collaboration beyond METROFOOD-RI's immediate community.

Finally, by articulating its service offer in a structured and transparent way, METROFOOD-RI strengthens its position for long-term sustainability. Stakeholders can clearly see the value provided across thematic areas of impact, supporting advocacy and strategic alignment with European research and innovation priorities.

## 5 Conclusions

The development of an updated service chart under the METROFOOD-EPI project has been a foundational step in enhancing METROFOOD-RI's role as a service-oriented European Research Infrastructure. The new service chart, structured into Research, ICT & Data, Advisory, and Education & Training services (with further subdivisions), provides a transparent overview of what METROFOOD-RI offers, from cutting-edge analytical measurements and reference materials to data tools, expert consulting, and capacity-building programmes.

The updated chart aligns METROFOOD-RI's services with key domains of impact, including agrifood innovation and safety, public health nutrition, environmental sustainability, and the underpinning of the metrology role on reliability of measurement results, standardization and harmonization. In doing so, it makes the infrastructure's contributions to societal challenges explicit and supports its integration into the broader landscape of research infrastructures and initiatives focused on food systems and open science. The inclusion of *ICT & Data services* and *Education & Training* as categories reflects current priorities in digital transformation and knowledge sharing.

Internally, the service chart serves as a framework for coordination and strategic planning, helping to identify gaps, avoid duplications, and foster cross-node collaboration. It also supports the organization of service information within the METROFOOD-RI membership database and contributes to strategic decision-making. Evaluation will follow an annual cycle using portal analytics (unique users, access requests, service usage), cross-node collaboration counts, post-access satisfaction scores on service discovery, and participation in training initiatives.

For external users, the chart will underpin the upcoming online access portal and catalogs, offering a user-friendly interface to browse and search METROFOOD-RI's services by categories. This is expected to improve service discoverability and increase user engagement. In the long run, the service chart can facilitate partnerships beyond METROFOOD-RI, even allowing joint service offerings with other infrastructures or by favoring integration with EOSC.

In conclusion, the METROFOOD-EPI service chart update has strengthened the foundation of METROFOOD-RI as a service-oriented infrastructure. It has translated the RI's multifaceted capabilities into a structured, comprehensive and accessible format, enabling the delivery of high-quality services in food and nutrition metrology, supporting scientific excellence, innovation, and public welfare. Moreover, the process and outcome provide a practical model for research infrastructures transitioning from preparatory to operational phases.

## Data Availability

The original contributions presented in the study are included in the article/Supplementary material, further inquiries can be directed to the corresponding author.
