# Supplementary material for: Advancing service offerings in food and nutrition metrology: the updated METROFOOD-RI service chart
Source: Front Nutr. 2025 Dec 18;12:1711663. doi: 10.3389/fnut.2025.1711663 (PMC12756099; doi:10.3389/fnut.2025.1711663)
Supplement: Supplementary file 1 [file Data_Sheet_1.pdf]

Annex I – Definition of service categories

Research services

Research services highlight one of the core businesses of METROFOOD-RI. It encompasses a broad range of services aimed at advancing knowledge, scientific excellence and innovation. It includes all experimental studies, analytical testing, and scientific evaluations. Research services are critical for driving scientific excellence, supporting evidence-based policymaking, and fostering technological and innovative advancements in food and nutrition. They are further grouped under four intermediate key categories:

- Agrifood
- Metrology tools
- Health
- Environment & sustainability

Agrifood

Covers services related to the entire food supply chain, from primary production to final consumption. It includes food production, processing, packaging, storage, quality, safety, and the development of novelty in foods and new materials & technologies. The agrifood sector is distinct due to its comprehensive coverage of all stages of food handling and its critical role in ensuring food safety, and quality for consumers. Its importance lies in maintaining the integrity and sustainability of the food supply chain, promoting innovation, and supporting cutting-edge research to improve agricultural practices and associated food technologies. Each of the sub-categories are further defined below.

Food production **-** Services related to the cultivation, harvesting, and primary production of food, covering experimental farms/field and greenhouse studies. It also involves plant growth experiments conducted under controlled climatic conditions, such as closed chambers, to enhance productivity and study environmental effects on plant health. Additionally, it includes the optimization of agroecosystem management practices to improve food composition, quality, and safety. Plant breeding is also encompassed, focusing on the development of new, improved plant varieties through traditional and advanced genetic techniques to enhance crop yield, resistance to pests and diseases, and adaptability to changing environmental conditions. The biotechnological production of food and ingredients, involving advanced techniques to enhance food quality and yield, also falls under this scope. Furthermore, it encompasses research and development in aquaculture to optimize the breeding and farming of aquatic species for food, as well as the optimization of livestock breeding practices to enhance the efficiency and sustainability of meat, dairy, and other animal product production. Differentiated by its focus on the initial stages of the food supply chain, it is critical for ensuring the sustainable and efficient production of raw food materials and aims to add resilience to the food chain.

Food processing, packaging & storage **-** Services that involve transforming raw food materials into consumable products, focusing on processing techniques, packaging innovations, and solutions. It includes pilot plants for testing and training (new) food processing and storage technologies, such as baking, dehydration, and high-pressure processing. Kitchen labs study domestic food preparation practices, including cooking and storage. The subcategory also covers the development of aids like sanitisers and food additives to enhance processing and storage. Furthermore, it encompasses the characterisation and safety testing of food contact materials, including overall and specific migration analysis, to ensure compliance with regulatory standards

and consumer safety. Additionally, it focuses on creating innovative packaging technologies, such as active and intelligent packaging, and strategies to reduce food losses through waste recycling and by-product valorisation. The development of smart sensors for processing, distribution, and storage is also key. This subcategory is distinct from food production as it deals with post-harvest activities that ensure food quality and shelf life.

Food quality, authenticity & traceability - Services focusing on the sensory and nutritional attributes of food products, including quality assessment, control measures, and certification processes to ensure consumer satisfaction and market competitiveness. It includes the evaluation of food quality through the analyses of bioactive compounds, physical-chemical properties, in vitro bioactivity, antioxidant activity, organoleptic properties, and microbiological assessments. The subcategory also covers food authenticity and traceability, using genetic markers, isotopic analysis, elemental profiling, metabolomics, and techniques to detect adulteration. Additionally, the service includes the development of new smart sensors to enhance the traceability and transparency of food systems, contributing to the health, safety, and sustainability of food products and processes. Furthermore, it addresses food fraud by employing advanced detection methods to identify and prevent the misrepresentation of food products, ensuring integrity within the food supply chain. The topic on nutritional quality is also specifically included as a subcategory within the health category to make sure that users with diverse backgrounds can easily navigate and find relevant services.

Food safety in production and supply chain - Services focusing on ensuring food safety through the comprehensive analysis of foods and the evaluation and identification of potential risks. It focuses on detecting, monitoring, and controlling contaminants, residues and additives throughout the food production and supply chain. This comprises services aimed at identifying and mitigating risks associated with, e.g., pesticides, toxic and potentially toxic elements, microplastics, nanomaterials, mycotoxins, additives, contaminants, pathogenic microorganisms, allergens, etc., which may arise during primary production, processing, packaging, or transportation. Services in this subcategory are aimed at maintaining compliance with safety standards, reducing risks at the source, and supporting agrifood stakeholders in producing safe products. The food safety subcategory is used in both the agrifood and health categorisations because it is relevant to users from various backgrounds. It further ensures that all users, regardless of their perspective or expertise, can easily find information and services they need to protect public health and ensure compliance with safety regulations.

Novelty in foods, new materials & technologies - Services addressing the development and evaluation of innovative food products (including novel foods but not limited to) and cutting-edge technologies in the agrifood sector. They are crucial for driving innovation, enhancing food security, and meeting the evolving demands of consumers. These advancements enable the creation of sustainable, nutritious, and safe food products, pushing the boundaries of traditional food production and processing methods. This subcategory is essential for fostering scientific breakthroughs and introducing transformative solutions to the food industry.

Metrology tools

Metrology in the context of food and nutrition involves services that ensure accurate and precise measurements in food analysis. This category includes the provision of Reference Materials, proficiency testing, and other metrological services that support the reliability of measurement results and comparability of data across laboratories and institutions. Metrology is essential for maintaining

high standards in food testing, ensuring consistency in analytical results, and supporting regulatory compliance and scientific research. Its distinct role in providing the foundational accuracy needed for all qualitative and quantitative assessments in the food sector makes it indispensable.

Reference Materials - Services focused on the development and production of Reference Materials (even customised) for calibration and quality control in analytical measurements. This category is important for ensuring the accuracy and comparability of measurement results in agrifood analysis. Homogeneity and stability studies are included as well.

Proficiency testings - Services focused in organising and managing inter-laboratory comparisons to assess the performance of laboratories. It is important for maintaining and improving the quality of laboratory analyses and ensuring consistency across different testing facilities.

Health

This category focuses on services related to the impact of hazardous chemicals, pathogens, particles, allergens and the potential negative effects of new technologies on consumer health. It includes food safety, risk assessment, health effects, and toxicology services. These health services are crucial for protecting public health, informing policy decisions, and ensuring that food products are safe for consumption. The unique focus on the intersection of food and health distinguishes this category, emphasising the direct impact of food on human well-being.

Nutritional quality & dietary insights - Services that evaluate the nutrient composition and balance of foods, including macronutrients (proteins, fats, carbohydrates) and micronutrients (vitamins, minerals). It emphasises the bioavailability of nutrients, their adequacy in supporting health, and their role in dietary patterns that promote well-being. Services in this category assess factors such as energy density, nutrient density, caloric content, and the impact of food composition and processing on long-term health outcomes like obesity, cardiovascular disease, and metabolic disorders. They are essential for understanding how foods contribute to dietary recommendations, prevent deficiencies, and support overall health.

Food safety and public health - Services aimed at ensuring food safety and protecting public health through analysis of foods and by identifying, assessing, and mitigating risks. It includes the assessment among other of toxins, allergens, heavy metals, pesticides, mycotoxins, pathogenic microorganisms, dioxins, microplastics, and other emerging risks. By focusing on toxicological assessments, exposure analysis, and risk characterisation, these services provide insights essential for protecting public health, supporting healthcare professionals, policymakers, and researchers in addressing foodborne risks.

Risk assessment - Services that evaluate potential health risks associated with food consumption, including hazard identification, dietary exposure assessment, and risk characterisation. These services are essential for informed decision-making in food safety and public health policies.

Health effects & Toxicology - Services that study the health impacts of food components and contaminants. It includes toxicological assessments, functional property evaluations (e.g., bioactive compounds, antioxidants, probiotics), nutritional studies, and evaluations of long-term health effects. Examples of technologies and methodologies used in this subcategory include bioassays, computational toxicology, in vitro testing, etc. Services in this category are essential for understanding the effects of various substances, such as additives, pesticides, heavy metals, naturally occurring toxins, and bioactive compounds on human health. Additionally, they assess

the impact of dietary patterns, novel food ingredients, and food processing methods on overall health.

Environment & Sustainability

Services that promote the sustainable management of natural resources and minimise the environmental impact of food production. It covers areas such as agroecosystem management, environmental monitoring, circular bioeconomy, and waste management. Services in this category are vital for ensuring that food production practices are environmentally friendly and sustainable, thereby ensuring that the negative impacts on ecosystems are minimised.

Agroecosystem management - Services focusing on the holistic and sustainable management of agricultural ecosystems. Services under this subcategory encompass practices such as soil health improvement, efficient water management, and biodiversity conservation. The primary aim is to enhance the sustainability and productivity of farming systems while maintaining ecological balance. This subcategory is distinct because it integrates various agricultural practices to create resilient agroecosystems that can withstand environmental stresses and support long-term food security.

Environmental monitoring - Environmental monitoring involves services that track and assess various environmental parameters related to food production. This includes monitoring air and water quality, soil health, and other environmental indicators. The focus is on gathering data to understand the environmental impacts of agricultural activities and ensuring that these activities comply with environmental regulations. Environmental monitoring is crucial for identifying potential environmental hazards, assessing the effectiveness of regulatory measures, and making data-driven decisions to protect the environment. Unlike agroecosystem management, which is proactive and integrative, environmental monitoring is more about ongoing assessment and compliance, providing the necessary measurements and data to guide sustainable practices and regulatory enforcement.

Circular bioeconomy - Services promoting the recycling and reuse of agricultural and food waste to create value-added products. They include comprehensive evaluation and pilot experiments to transform agrifood by-products into valuable food ingredients. Additionally, they emphasise the importance of detecting both nutrients and contaminants using validated procedures and metrology practices, supporting a shift from the traditional linear economy to a circular approach that enhances resource efficiency, minimises waste, and advances sustainability in the food industry.

Waste management - Waste management services involve the proper disposal, treatment, and handling of agricultural and food waste. This subcategory includes the development of strategies for reducing food losses and managing food surplus through waste recycling and the valorisation of by-products. Waste management is crucial for preventing the negative impacts of waste on ecosystems and human health, focusing on the safe and efficient disposal of waste materials and reducing the immediate environmental footprint of food production and consumption activities.

ICT & data services

This category includes services offered in the e-RI related to data, such as tools, software and datasets.

Existing software & repositories **-** Services that provide access to existing, specialised software tools and data repositories available within METROFOOD-RI facilities. It offers resources that are immediately accessible and standardised across the infrastructure. Existing software services will provide existing specialized tools and software for data analysis, visualisation, and management (e.g., managing data from laboratories). Existing data collections and repositories will support the collection of high-quality data and datasets related to metrology and food, such as food consumption data, as well as data repositories where user can search and retrieve data.

Software development **-** Services that provide development of a completely new software created according to the user's requirements.

Data repository development **-** Services that provide development of new databases and repositories, including services that support the collection of high-quality data, e.g., if the user would like to have an easily accessible database of all their laboratory results in one place.

Website development **-** Services that provide development of completely new websites created according to user's requirements.

Data analysis **-** Services helping with the analysis of data.

Hosting **-** Hosting of services on servers.

Computing **-** Provision of access to a high-powered computing, e.g., if the user had a lot of calculations that required high-powered computing.

Virtual research environments - Creation of virtual research environments, e.g., remote virtual desktop.

Advisory services

This category offers expert advice, technical support, and guidance to stakeholders in agrifood and nutrition sector. It includes services related to regulatory compliance, strategic planning, and technical problem-solving. Advisory services are crucial for helping organisations navigate complex regulatory environments, improve operational efficiency, and implement best practices.

Regulatory compliance **-** Services aimed at helping organisations comply with national and international food safety and quality standards and regulations. This category is vital for ensuring that food products meet legal standards and avoid regulatory penalties.

Expertise & Support **-** Services that provide stakeholders with specialised knowledge, technical assistance, and strategic advice to enhance their operations, drive innovation, and address specific challenges in all METROFOOD-RI areas, such as food production and processing, food safety, quality control, sustainability, etc. It is important for providing specialised knowledge and assistance to help stakeholders understand and navigate the complex landscapes within the agrifood sector.

Education & Training

This category encompasses a wide range of educational and training services aimed at building competencies and knowledge. It includes formal and informal learning opportunities which are

essential for developing the skills and expertise needed to advance research, innovation, and best practices in food and nutrition.

Laboratory and technical training - Hands-on training in a wide range of laboratory techniques and technical skills. It includes practical instruction on the use of laboratory equipment, experimental procedures, data analysis, and adherence to safety and quality standards. Laboratory and technical training are crucial for building the competencies of researchers, technicians, and students, ensuring they are proficient in modern laboratory practices and capable of conducting high-quality research and analysis.

Webinars - Virtual educational sessions that allow participants to join remotely via the internet. It comprises live presentations, demonstrations, or lectures, often with interactive elements such as Q&A sessions, polls, and chat features, thereby providing a flexible and accessible platform for disseminating information to a broad audience regardless of geographical location.

Seminars - Seminars on a single topic or a few closely related topics, typically involving presentations by one or a few experts, followed by discussions and Q&A sessions. This will foster a more interactive and personalised learning experience, making them ideal for specialised training, advanced education, and collaborative problem-solving.

Conferences - Organisation and contributions to large-scale events focusing on a wide range of topics in Food & Health, including public and networking events.

Workshops - Workshops are interactive sessions designed to provide practical training and facilitate knowledge sharing on food-related topics. This can involve hands-on activities, group discussions, and collaborative problem-solving exercises.

Online courses - Structured learning programmes delivered virtually via the internet, designed to educate participants on various topics. Courses can range from short, self-paced modules to comprehensive, instructor-led programmes spanning several weeks or months. Online courses can include a combination of video lectures, readings, interactive activities, assessments, and discussion forums.

Internships - Practical work experience, allowing students and early-career professionals to apply their know-how in real-world settings. Internships designed to train the next generation of professionals, offering hands-on experience in laboratories, research institutions, and industry settings associated with METROFOOD-RI.

Summer/Winter schools - Intensive, short-term educational and networking programmes focusing on topics in the food and nutrition. Summer/winter schools are important for providing in-depth knowledge on innovative technologies and best practices and offering networking opportunities.

Study visits - Organised visits to research institutions, laboratories, and companies associated with METROFOOD-RI to learn about best practices and innovative technologies. Study visits are crucial for knowledge exchange, human resource upskill and fostering partnerships.

Integrated services

The following paragraphs present in-depth elaborations on each of the six integrated services; however, they are intended to be illustrative rather than exhaustive and are aimed to visualise service

examples throughout all NNs. Additionally, training and technology transfer will be made available across all services, ensuring comprehensive support for each offering.

Traceability, authenticity and transparency in the food chain

The authenticity detection and traceability of several foods is fundamental to safe consumption of food, as well as to counter issues regarding fraud and adulteration. Falsification in geographical origins of food, food content indication on labels in packaging, misbranding are a few examples of common food-related concerns. Enhanced methods to counter issues of authenticity, traceability and transparency will be necessary to prevent food fraud, protect regional and organic food brands/growers and to comply with established trade regulations. The integrated service combines the following solutions (which belong to specific services falling under the categories shown in **Figure A1**, together forming the integrated service):

- Development of advanced food authenticity protocols involving innovative approaches (genetic markers, multi-methods, etc.)
- Omics analysis of food for assessment of safety, adulteration, origin, and authenticity.
- Implementation of tailored blockchain solutions in support to the development of integrated traceability systems along specific food chains
- Implementation and application of AI algorithms to support traceability, authenticity and origin demonstration and identification of food frauds
- Traceability Search Engine

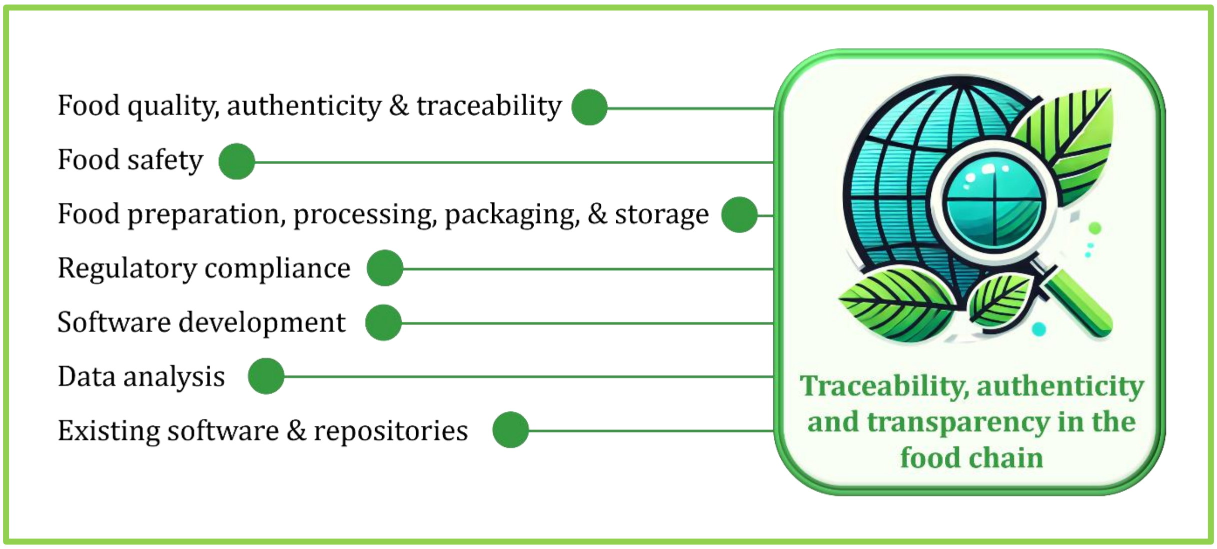

Figure A1 *- Service categories included to build the integrated service on traceability, authenticity, and transparency in the food chain.*

The National Nodes contribute to advancing ***traceability, authenticity, and transparency in the food chain*** by combining their unique capabilities and expertise in omics approaches, advanced analytical techniques, and digital solutions. Germany and Czech Republic are engaged in omics analysis, which supports the assessment of food safety, origin, authenticity, and potential adulteration. Turkey is developing a blockchain-based traceability platform under the TARIM2030 project, which integrates climate and physiological monitoring, production controls, certification, and sustainability metrics into a unified data model, thereby ensuring transparency and quality in the food production process. Similarly, Italy and Spain focus on advanced food authenticity protocols, employing innovative

approaches like genetic markers and multi-method analyses. Italy also has specific expertise in implementing blockchain and AI technologies to support integrated traceability systems and food fraud identification. Portugal and North Macedonia contribute through multi-elemental analysis and fatty acid profiling, supporting chemical fingerprinting for food authentication. Greece also adds to the authenticity toolkit with untargeted and targeted metabolomics/lipidomics using advanced spectroscopic techniques (UHPLC-HRMS, UPLC-MS/MS, NMR, GC-MS/MS) and spectral fingerprinting (UV-Vis, FT-MIR) to analyse and verify the authenticity of food products against reference libraries. Additionally, Slovenia specializes in Internet of Things (IoT) systems and olive oil traceability, integrating digital tools and analytical methods to ensure transparent and sustainable food production chains. Romania has expertise in supporting comprehensive assessments of food authenticity and traceability. Switzerland, on the digital front, develops ICT specifications, acting as a liaison between scientific fields. Their node provides a traceability search engine to visualize data across the food supply chain and can implement AI-based applications to analyse food fraud patterns. The production of reference materials by Germany, Belgium, Italy, and Romania are also crucial for standardisation in authenticity verification.

Emerging food risks

Emerging contaminants (such as PFAS, micro- and nano-plastics, toxins, etc.) continue to be prevalent in food systems and may pose health threats. They may be present throughout the food production and supply systems. Monitoring and their control at critical points become even more necessary with regards to consumption of safe food. The integrated service combines the following solutions (which belong to specific services falling under the categories shown in **Figure A2**, together forming the integrated service):

- Enhanced method development for emerging contaminants and their detection
- Advanced analytical techniques and contribution to their study in specific conditions (low-levels, precision detection, novel/new food matrices)
- Development of new matrix-Reference Materials
- Capacity building (training, info sessions, etc.) for the detection and analysis of emerging contaminants among researchers and laboratory experts in the EU
- Exposure and risk assessment

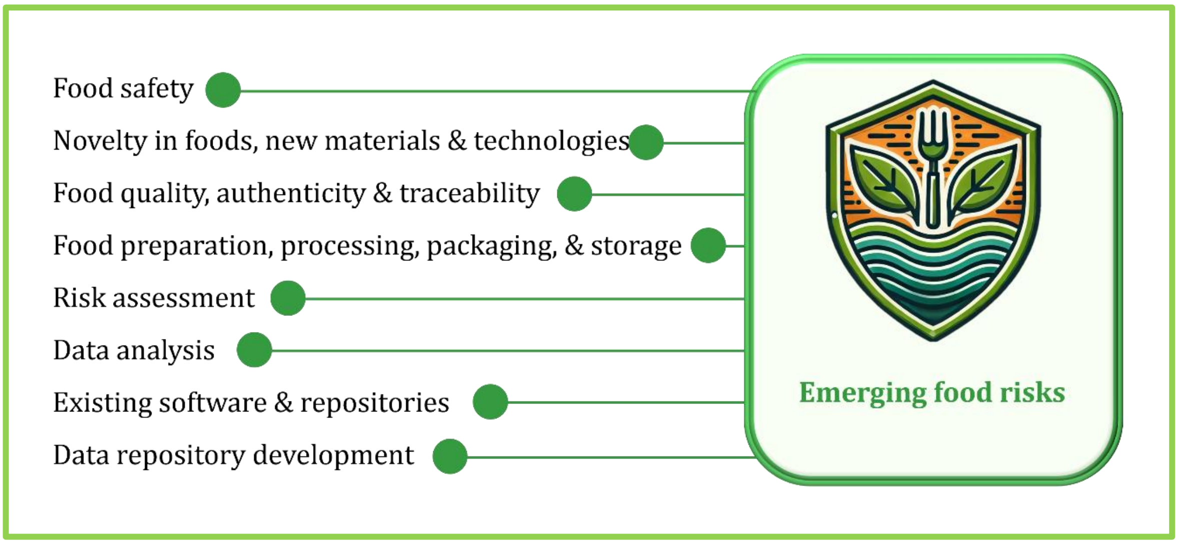

Figure A2- *Service categories included to build the integrated service on emerging food risks.*

To address ***emerging food risks***, NNs collectively leverage diverse capabilities across Europe. Germany, Italy, Belgium, Spain, and the Czech Republic focus on the development of new methods and the application of advanced analytical techniques for detecting emerging contaminants, including those requiring low-level and precision detection in novel food matrices. Additionally, Romania, Portugal, Greece, Turkey and North Macedonia specialize in detecting and quantifying chemical, microbiological, and allergenic hazards in food products. Belgium, Greece and North Macedonia, in particular, provide targeted and non-targeted screening of contaminants like pesticide residues and substances migrating from food contact materials. Supporting standardised testing across the infrastructure, facilities in Italy, Germany, Belgium, Turkey, and Romania are actively involved in the creation of new matrix Reference Materials (RM), which are essential for ensuring consistent and reliable analysis. Together with Slovenia and Switzerland, they have further expertise in exposure and risk assessment, critical components in managing food safety. Switzerland plays a key role in digital support for risk assessment, managing databases and apps such as FoodCASE that link contaminant data to food consumption information, facilitating a comprehensive approach to exposure assessment. Switzerland further supports the infrastructure by gathering ICT requirements, acting as a liaison between chemists, food scientists, and computer scientists, and developing data repositories for contaminant information. Switzerland also manages the METROFOOD RM app and oversees other digital tools that enable exposure assessment, fostering a coordinated and efficient approach to data management.

Novelty in foods, food additives & ingredients and alternative food systems

Novelty in foods and food ingredients, new food additives, novel foods, alternative food systems (including alternative protein sources) are fast becoming mainstream agents in the food and nutritional landscape in Europe and around the world. At the same time, such products are highly revered due to the potential contribution to a circular economy. Therefore, specific monitoring and analytical techniques, method development and regulatory guidance on such substances become even more important given their prevalence in food and feed. In this regard, an integrated set of services will provide expertise in their detailed analyses, using state-of-the-art technologies and expert guidance via

the METROFOOD-RI facility. The integrated service combines the following solutions (which belong to specific services falling under the categories shown in **Figure A3**, together forming the integrated service):

- Use of alternative protein sources and their safety determination
- Analysis of ingredients for such novel or new food or food additives, including foods containing nanotechnologies
- Investigation of the methods of production and processing (recipes) of novel food and food additives
- Implementation of dedicated e-tools, such as:
    - Alternative Protein Source Database
    - Extended Gamma-Wiki
    - Protein content comparison App
    - Recipe AI App

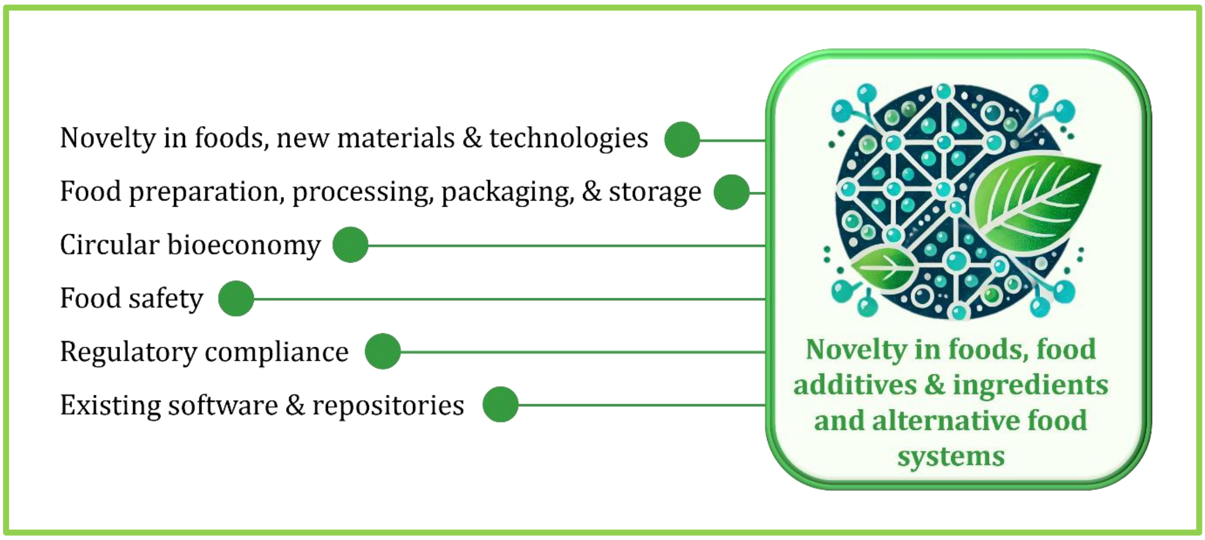

Figure A3 - *Service categories included to build the integrated service on novelty in foods, food additives & ingredients, and alternative food systems.*

To contribute significantly to research and development in ***novelty in foods, food additives & ingredients, and alternative food systems***, Germany and Belgium have experience in the analysis of ingredients, food additives and alternative protein sources, while North Macedonia can analyse contaminants, food additives and determine the nutritive composition of food. Romania is well-equipped to support the development of alternative protein sources, functional additives, and plant-based systems, utilizing advanced mass spectrometry and spectroscopic techniques for ingredient analysis, while Greece focuses on the biotechnological production of natural carotenoids like β-carotene and lycopene, used in food and feed products, advancing sustainable natural additives. Slovenia brings expertise in the characterization of the quality and safety of food products while Turkey coordinates a TWINNING project to enhance excellence in plant protein research, creating a network that strengthens research and innovation in plant-based protein, benefiting from omics platforms and best practices exchange. Portugal also provides characterization of food ingredients, focusing on nutritional value and contaminant analysis, alongside genomic analysis of nutrient impact on cells and tissues. Spain contributes through health and nutritional profiling and omics analysis, while Italy supports ingredient analysis and the development of novel foods, with expertise in

growing insects and algae, and facilitates the implementation of e-tools for managing food-related data. Switzerland aids in digital infrastructure by gathering ICT requirements and developing a data repository for protein data, including apps like the Protein Content Comparison App and Recipe AI App, linking them to relevant data sources for comprehensive integration. The Czech Republic contributes with its facilities for rearing edible insects, providing nutritional evaluation and supporting the Alternative Protein Source Database with data on edible insects. As previously mentioned, facilities in Italy, Germany, Belgium, and Romania can create new matrix RMs, ensuring consistent and reliable analysis.

Innovative food processing

Consumer trends and demands show a rising increase in food that contain no or minimal additives. Food processing requires a number of food additives for multiple reasons – improved shelf-life, pleasant aromas, colour, texture and so on. However, food processing technologies could be improved to minimise the use of additives thereby catering to the growing demand of consumers for low-additive food products. The integrated service combines the following solutions (which belong to specific services falling under the categories shown in **Figure A4**, together forming the integrated service):

- Explore food processing alternatives and their safety profiling of natural food additives (e.g., natural carotenoids focusing on β-carotene and lycopene)
- Demonstrative pilot for minimising/avoiding food processing contaminants
- Research, demonstration and training on sustainable and integrated downstream processes finalised to extraction of functional molecules from biological matrices

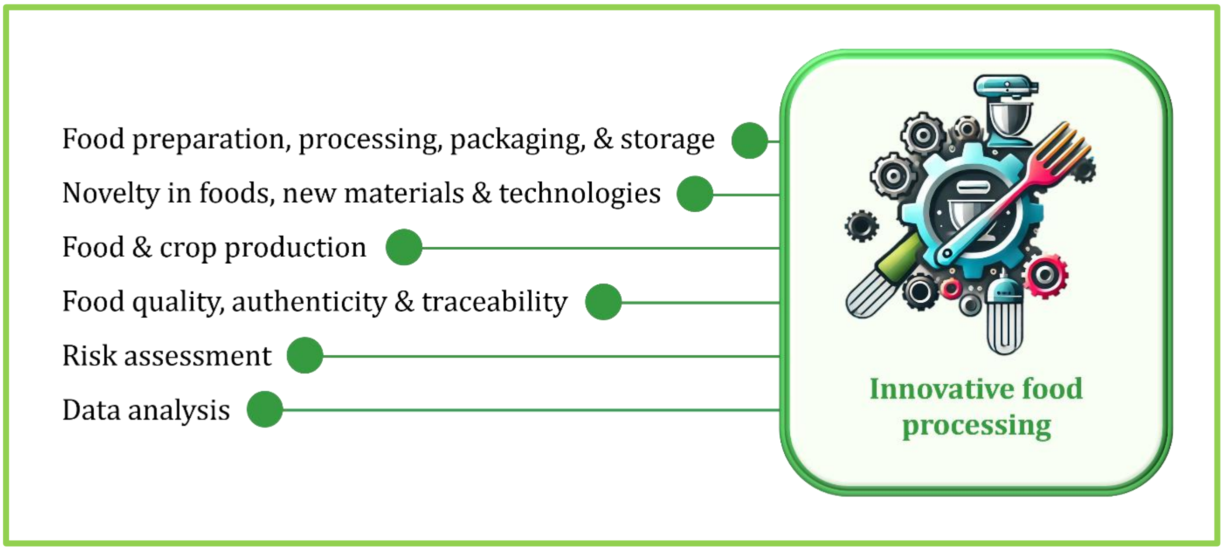

Figure A4 *- Service categories included to build the integrated service on innovative food processing.*

The NNs demonstrate diverse expertise in **innovative food processing**, with Germany exploring alternatives in food processing, examining new approaches to address emerging challenges in the food industry. Turkey offers a pilot facility for processing projects, including the transformation of fruit residues into food supplements, showcasing its capability to repurpose food by-products for added value. Portugal focuses on characterising food products before and after processing, assessing

nutritional value, contaminants, and bio-accessibility. They also conduct genomic analyses to understand how contaminants and nutrients impact cells and tissues, adding a molecular perspective to food processing studies. Facilities in Spain support safety profiling of natural food additives, such as carotenoids (e.g., β-carotene and lycopene). These facilities also offer pilots for minimizing processing contaminants and provide research, demonstration, and training on sustainable processing methods that extract functional molecules from biological sources. Slovenia brings expertise in encapsulation technologies, which play a crucial role in protecting active ingredients, improving stability, and controlling the release of functional compounds in food products. Italy contributes with monitoring systems for food processing performance, particularly in sustainable downstream processes, enabling the extraction of valuable functional molecules from biological matrices. Italian labs also conduct quality and safety testing and support research and training initiatives on integrated food processing alternatives. Romania has extensive facilities for pilot-scale testing of innovative processing technologies. They conduct R&D on both thermal and non-thermal methods to improve product quality, safety, and shelf life. The facilities support the full cycle from design and characterization to technology optimization and product development at pilot scale. Once again, Switzerland supports these efforts by collecting ICT requirements, while Czechia operates a food processing pavilion with a focus on dairy, meat, and bakery technologies. FRIP specifically works on high-pressure treatment for food preservation, advancing non-thermal processing methods. Belgium, meanwhile, concentrates on the safety profiling of natural food additives, ensuring that these innovations meet regulatory and safety standards.

New generation packaging solutions

Recent trends have seen a significance development in the field of food packaging, where more ‘green’ and ‘clean’ packaging are considered when it comes to sustainable food packaging. Innovations in food packaging have given rise to several new generation packaging materials, derived from non-traditional sources including bioactive sources that may impact the food packaged inside them. Consequently, monitoring and reporting on (un)intentional migration of any particles from such packaging solutions becomes imperative from a wider food safety perspective. The integrated service combines the following solutions (which belong to specific services falling under the categories shown in **Figure A5**, together forming the integrated service):

- Development of new bioactive and biobased packaging and intelligent packaging
- Testing of the suitability of the packaging as a food contact material, exploring its safety and stability
- Exploration of the capability of such packaging solutions in increasing the shelf-life of food products
- Investigation and characterisation of particles and their potential release such as micro-/nano-plastics from such packaging
- Investigation on the impact of these substances on food quality, texture and composition.
- Implementation of dedicated sensors, QR-codes or other intelligent labels to be incorporated in the new packaging solutions
- Technology transfer initiatives addressed to food packaging producers and food producers

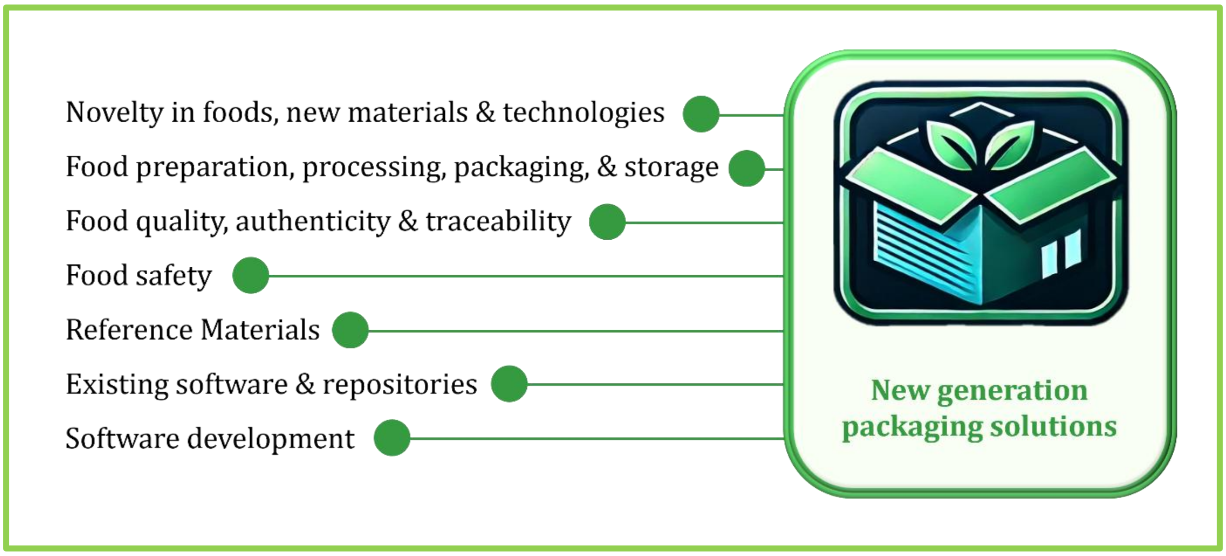

Figure A5 *- Service categories included to build the integrated service on new generation packaging solutions*

The range of expertise offered for ***new generation packaging solutions***, focuses on testing, safety, and intelligent packaging features to enhance food preservation and sustainability. Germany contributes by evaluating the impact of packaging on food quality, while Portugal specializes in global migration studies and explores intelligent packaging solutions. Facilities in Spain test packaging suitability as a food contact material, focusing on safety and stability, and investigate the release of micro- and nano-plastics from packaging materials through their Omics Sciences facility. Slovenia adds significant expertise with the development of new active and intelligent packaging, edible barcodes for traceability and authenticity, microbiological safety testing, and chemical safety evaluation, demonstrating a holistic approach to enhancing packaging functionality and safety. Belgium also specifically focusses on the release, safety and risk assessment of chemicals from food contact materials and the physiochemical characterisation of micro- and nanoparticles from food contact materials and their release into food. Facilities in Italy are involved in developing and testing new packaging, including assessments on shelf-life extension, micro- and nano-plastic release, and technology transfer to industry. Romania also provides significant support by evaluating the physical and chemical properties of innovative packaging. It further hosts the only accredited lab in Romania for migration testing in food simulants and works on incorporating smart features such as sensors and QR codes into packaging. Greece contributes by performing targeted and non-targeted screenings for substances migrating from food contact materials, ensuring packaging safety. Switzerland's role focuses on ICT support, gathering requirements and developing tools for simulating packaging impacts on foods and creating a database for packaging-related data. Czech Republic can contribute through research on encapsulation and release technologies of plant-based natural preservatives, which may be relevant for extending the shelf life of packaged foods.

Valorisation of side streams for circular bioeconomy approaches in agrifood

Valorising food production side streams is key to building a sustainable circular bioeconomy. METROFOOD's integrated services focus on transforming by-products and waste into valuable resources, promoting sustainability and reducing environmental impact. The integrated service

combines the following solutions (which belong to specific services falling under the categories shown in **Figure A6**, together forming the integrated service):

- Extraction and validation of high added-value molecules from by-products and side streams
- Systemic approach development of recovery processes aimed at achieving zero-residue outcomes
- Life cycle assessment
- Analytical services to characterise the new ingredients and final food products
- Development of e-tools such a database on by-products or recipes
- Recipe, process and production development to help industries convert food by-products into high-value materials.
- Safety and quality assurance for products derived from side streams, ensuring compliance with food standards

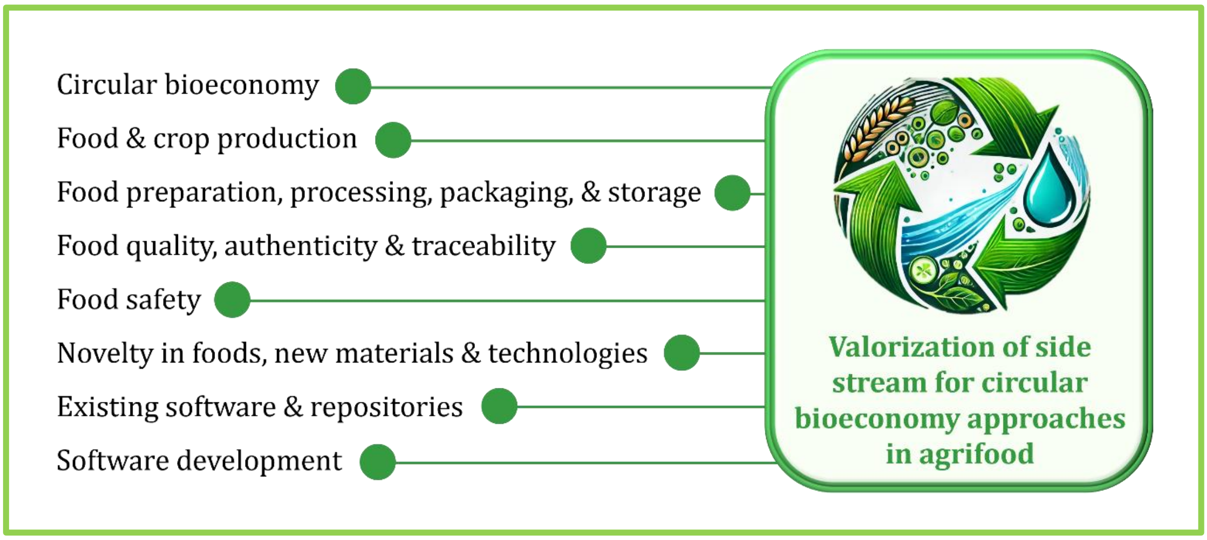

Figure A6 *- Service categories included to build the integrated service on valorisation of side streams for circular bioeconomy approaches in agrifood.*

For the ***valorisation of side streams for circular bioeconomy approaches in agrifood***, Germany is involved in the extraction of high-value molecules, providing analytical services for characterization and quality assurance, as well as developing e-tools, such as databases on by-products and recipes, to support the valorisation process. Turkey operates the "smart applications research centre for food supply security," focusing on alternative fertilization techniques, data analytics for controlled agriculture, and developing resilient crops suitable for extreme environments. This node also explores the use of smart technologies to assess the impact of various agricultural practices on crop quality and food safety. Portugal contributes to food characterisation of side stream products, assessing both nutritional value and contaminants, along with conducting bio accessibility studies to evaluate the effectiveness of these by-products. In Spain, facilities such as the EUT nutrition and health, omics sciences, and sustainability labs are engaged in the extraction of high-value molecules, systemic recovery processes aiming for zero waste, life cycle assessments, and the creation of e-tools to catalogue by-products and develop recipes. Together with Slovenia, these facilities also assist industries in converting food by-products into high-value materials while ensuring product safety and quality. Italy brings specialised techniques for the extraction of valuable compounds from by-products, utilizing processes like membrane filtration and supercritical CO_2 extraction. Italian labs are

also involved in the characterisation of ingredients, life cycle assessments, and the development of databases to track by-products and recipes. Romania further supports this effort by analysing side streams from food production, assessing their potential for transformation into products like biofuels, animal feed, and biodegradable materials, with access to pilot plants for experimental processing. Switzerland plays the digital support role by gathering ICT requirements, mediating between scientists and technologists, and managing tools like the FoodCASE databases which can be linked with FoodWasteExplorer from Slovenia. They aim to link data on by-products and waste materials to create a more comprehensive resource for valorisation. Czechia focuses on agricultural production, with an interest in primary processes that can utilize side streams. Belgium can provide services for ensuring the safety and quality of products derived from side streams, supporting compliance with food standards, which is essential for market acceptance and consumer safety.

The integrated services will keep evolving over time and will include additional services based on emerging needs and demands in food and nutrition, with the possibility to be tailored time by time to the actual demand and user needs.
